# Stress response during early sedation with dexmedetomidine compared with usual-care in ventilated critically ill patients

**DOI:** 10.1186/s13054-022-04237-0

**Published:** 2022-11-22

**Authors:** John P. R. Moore, Yahya Shehabi, Michael C. Reade, Michael Bailey, John F. Fraser, Lauren Murray, Christopher Anstey, Mervyn Singer

**Affiliations:** 1 Department of Intensive Care, Sunshine Coast University Hospital, 6 Doherty St, Birtinya, QLD 4575 Australia; 2 Monash Health School of Clinical Sciences, Monash University, Melbourne, Australia; 3 Faculty of Medicine, University of Queensland, Brisbane, Australia; 4 Australian and New Zealand Intensive Care Research Centre, Monash University, Melbourne, Australia; 5 Critical Care Research Group, University of Queensland and The Prince Charles Hospital, Brisbane, Australia; 6 School of Medicine, Griffith University, Southport, QLD Australia; 7 Bloomsbury Institute of Intensive Care Medicine, University College London, London, UK

**Keywords:** Allostasis, Critical illness, Multiple organ failure, Sedatives

## Abstract

**Background:**

Sedative agents may variably impact the stress response. Dexmedetomidine is a sympatholytic alpha_2_-adrenergic agonist mainly used as a second-line sedative agent in mechanically ventilated patients. We hypothesised that early sedation with dexmedetomidine as the primary agent would result in a reduced stress response compared to usual sedatives in critically ill ventilated adults.

**Methods:**

This was a prospective sub-study nested within a multi-centre randomised controlled trial of early sedation with dexmedetomidine versus usual care. The primary outcome was the mean group differences in plasma levels of stress response biomarkers measured over 5 days following randomisation. Other hormonal, biological and physiological parameters were collected. Subgroup analyses were planned for patients with proven or suspected sepsis.

**Results:**

One hundred and three patients were included in the final analysis. Baseline illness severity (APACHE II score), the proportion of patients receiving propofol and the median dose of propofol received were comparable between groups. More of the usual-care patients received midazolam (57.7% vs 33.3%; *p* = 0.01) and at higher dose (median (95% interquartile range) 0.46 [0.20–0.93] vs 0.14 [0.08–0.38] mg/kg/day; *p* < 0.01). The geometric mean (95% CI) plasma level of the stress hormones, adrenaline (0.32 [0.26–0.4] vs 0.38 [0.31–0.48]), noradrenaline (4.27 [3.12–5.85] vs 6.2 [4.6–8.5]), adrenocorticotropic hormone (17.1 [15.1–19.5] vs 18.1 [15.9–20.5]) and cortisol (515 [409–648] vs 618 [491–776)] did not differ between dexmedetomidine and usual-care groups, respectively. There were no significant differences in any other assayed biomarkers or physiological parameters Sensitivity analyses showed no effect of age or sepsis.

**Conclusions:**

Early sedation with dexmedetomidine as the primary sedative agent in mechanically ventilated critically ill adults resulted in comparable changes in physiological and blood-borne parameters associated with the stress-response as with usual-care sedation.

**Supplementary Information:**

The online version contains supplementary material available at 10.1186/s13054-022-04237-0.

## Introduction

The stress response is a fundamental biological mechanism that has evolved to allow human beings to respond to both internal and external stimuli. These include events associated with daily living such as exercise and arguments, but also pathological events such as illness or injury. It is coordinated by the primitive brain structures of the diencephalon and brainstem in response to somatosensory inputs, and comprises a broad range of haemodynamic, respiratory, metabolic, neuro-hormonal, immune and behavioural effects [1]. Stress imparts an allostatic load on the body but, in health, this is adaptive and of appropriate degree. Downstream physiological and behavioural changes compensate to maintain overall stability and then normalise once the stress has passed. In disease states, especially if severe and/or prolonged, the body can enter a maladaptive state of allostatic overload that may contribute directly to the pathophysiology of the illness [2]. This is exemplified by the chronic stress-related diseases of hypertension, stroke, obesity and metabolic syndrome [3] and, more acutely, Takotsubo cardiomyopathy [4]. The metabolic shutdown seen in cases of severe critical illness and shock may represent an adaptive response of end-organs to severe stress to preserve their long-term survival at the expense of short-term, higher levels of function observed clinically as multi-organ dysfunction/failure [5, 6].

This sub-study of the SPICE III trial examined the effects of sedation using the selective alpha-2 adrenergic agonist and sympatholytic agent, dexmedetomidine against usual-care using propofol and/or midazolam on physiological and blood-borne markers of different limbs of the efferent stress system in critically-ill patients.

The safe delivery of intensive care often necessitates use of sedative medications, however the optimal strategy in terms of sedative agent and depth of sedation remains uncertain. Gamma-aminobutyric acid (GABA) receptor agonists such as propofol or midazolam [7] and, more recently, the selective alpha-2 adrenoreceptor agonist, dexmedetomidine, are the most commonly used sedative agents in a critical care context. Dexmedetomidine provides sedation and analgesia without impacting respiratory drive. When compared to other agents, it results in shorter ventilation times [8] and a reduced incidence and duration of coma and delirium [9, 10]. As a sympatholytic agent, dexmedetomidine exerts effects on a major limb of the stress response [11]. Dexmedetomidine may also modulate other efferent limbs of the stress response. Early use of dexmedetomidine could potentially alter the evolution of organ failure and outcomes from critical illness in a manner distinct from GABA-agonist sedatives.

In this sub-study, performed in a subset of trial participants of the Sedation Practice in Intensive Care Evaluation (SPICE-III) trial [12], we determined whether early sedation using dexmedetomidine compared to GABA agonists results in an altered stress response. We also sought to determine whether GABA agonists adversely affected the normal coordination functions and efferent haemodynamic, respiratory, metabolic, neuro-hormonal, immune and signals of the central stress centres in the brain to a greater degree than dexmedetomidine.

## Materials and methods

This was a prospective, parallel group, longitudinal, multi-centre sub-study of the SPICE-III trial. The SPICE-III trial was a randomised, open-label trial in which critically ill adults requiring mechanical ventilation received early sedation with dexmedetomidine as the sole or primary agent, or usual-care with the physician’s choice of propofol, midazolam and/or other agents to determine the effect of sedation strategy on patient centred outcomes (Additional file 1: Appendix Figs. S1 and S2). Patients were included if they were aged 18 years and over, had been commenced on mechanical ventilation within 12 h of randomisation, and were expected to require mechanical ventilation and sedation for longer than one calendar day (Additional file 1: Appendix Table S1).

We hypothesised that a light sedation level (a Richmond Agitation-Sedation Scale (RASS) of – 2–1 [13]) achieved using dexmedetomidine compared to other sedative medications would result in differences in physiological and biochemical stress markers over 5 days. Feasibility was determined by a single centre pilot study [14].

This sub-study was conducted in four Australian ICUs from January 2017 until February 2018. Exclusion criteria were identical to the SPICE-III study. The study was registered at ANZCTR.org.au (identifier: ACTRN12616001200471) and approved by the Prince Charles Hospital Ethics Committee (HREC/16/QPCH/141). Prior consent or consent to continue in the trial was obtained from all patients or their proxies according to local regulatory requirements.

Following randomisation to the trial, physiological data, relevant drug dosing and blood-samples were collected at the time of randomisation (day 0) and at 0800 on days 1, 3 and 5 following randomisation. Sedation index was calculated as the sum of negative Richmond RASS measurements divided by the total number of assessments [15]. Patients who died before day 5 were included in the final analysis, provided consent had been given.

### Physiological measurements

Physiological data were collected as representations of metabolic, cardiovascular and respiratory stress. Research staff retrospectively reviewed the medical record and collected the temperature, heart rate, mean arterial pressure, respiratory rate and minute volume as recorded by bedside nursing staff. Single data points were taken at times which corresponded to blood sampling.

### Biochemical measurements

Plasma was assayed for levels of hormones and markers which are traditionally accepted to respond to stressful stimuli:

(i) Stress hormones (noradrenaline, adrenaline, aldosterone, ACTH and total cortisol),
(ii) Anabolic and catabolic hormones including free triiodothyronine (FT3), thyroxine (T4), thyroid stimulating hormone (TSH), growth hormone (GH), leptin, insulin, testosterone, oestradiol and prolactin.
(iii) Markers of lipid metabolism (triglycerides, total cholesterol and high-density lipoprotein cholesterol (HDL), beta-hydroxybutyrate)
(iv) Markers of cardiac injury and dysfunction (troponin I (TnI), brain natriuretic peptide (BNP)).

Laboratory results for glucose, urea, creatinine, alanine transferase (ALT), aspartate transferase (AST), alkaline phosphatase (ALP), albumin, pH, base excess (SBE), lactate, white cell count (WCC), platelets (Plt) and prothrombin time (PT) were collected from the medical records at times corresponding to the blood draws. Demographic data and patient-centred outcomes were obtained from the SPICE-III database.

### Biochemical methods

Levels of FT3, T4, testosterone and cortisol were measured using a competitive binding chemiluminescent immunoenzymatic assay (Beckman Coulter Synchron Clinical Systems®,, CA, USA) Oestradiol was measured by chemiluminescent immunoenzymatic assay ((DiaSorin Liaison® XL, Saluggia, Italy). Urea, ALP, ALT and AST were measured by the enzymatic rate method (Beckman Coulter). Aldosterone was measured by liquid chromatography-tandem (Beckman Coulter). TSH, prolactin, insulin, TnI and BNP were measured by non-competitive binding chemiluminescent immunoenzymatic assay. (Beckman Coulter). Leptin was measured by radioimmunoassay (Merck, Burlington, MA, USA). Adrenaline and noradrenaline were measured by reverse phase isocratic high-performance liquid chromatography. ACTH and GH were measured by solid-phase, two-site sequential chemiluminescent immunometric assay (Immulite® 2000 XPi, Siemens, Erlangen, Germany). Total cholesterol, triglycerides, HDL, beta-hydroxybutyrate and albumin were measured by the spectrophotometric timed-endpoint method (Beckman Coulter).

### Outcomes

The main study outcomes were alterations in level of markers associated with the different limbs of the efferent stress system (stress hormones, anabolic and catabolic hormones, lipid metabolism, acute-phase reactants and markers of cardiac injury or dysfunction, organ function indices, and physiological parameters). The primary analyses are comparison between dexmedetomidine versus usual care with propofol or midazolam. Sepsis represents a discrete pathophysiological subtype of critical illness, accordingly a secondary analysis of stress biomarkers in the septic subgroup was performed.

As the post hoc subgroup of patients in the SPICE-III trial above the age of 63.7 years demonstrated a lower mortality with early sedation with dexmedetomidine, we performed a post hoc analysis the effect of sedation strategy on stress biomarkers on a similar subgroup in this study.

### Statistical analysis

With a minimum of 50 patients per group, this study had > 90% power (two-sided *p* value of 0.01) to detect a difference in any given biomarker equivalent to 80% of one standard deviation. A difference of this magnitude equates to an approximate 20% change across the range of the marker and is perceived to be of clinical importance.

All data were assessed for normality and log-transformed where appropriate. Baseline comparisons were performed using chi-square tests for equal proportion, Student’s t test was used for normally distributed data, and Wilcoxon rank sum tests for nonparametric data with results reported as *n* (%), mean (standard deviation) or median (interquartile range), respectively. Longitudinal comparisons over time were determined by repeated measures analysis of variance. Longitudinal results are reported as least square means (95%CI) or geometric means (95%CI) where data were well approximated by a log-normal distribution. To account for a baseline imbalance in age, a sensitivity analysis was performed on all markers adjusting for patient age as a covariate. Analysis was performed using SAS version 9.4 (SAS Institute Inc., Cary, NC, USA). To increase the robustness of findings, a two-sided *p* value of 0.01 was used to indicate statistical significance.

## Results

### ***Patient characteristics ***(Table 1)

**Table 1:** Demographic and Clinical Characteristics of the Patients at Baseline

	Dexmedetomidine (*n* = 51)	Usual care (*n* = 52)
Age at randomisation (y), Mean (SD)	61 (15.9)	66.6 (10.5)
Male sex, *n* (%)	30 (58.8)	33 (63.5)
Weight (kg), Mean (SD)	86.5 (20.2)	90.3 (20.7)
APACHE II score pre-randomisation, Mean (SD)^§^	20.3 (7.91)	21.5 (6.91)
Sepsis, *n* (%)	33 (64.7)	33 (63.5)
Admission source		
Emergency department, *n* (%)	16 (31.4)	20 (38.5)
Hospital floor/ward, *n* (%)	12 (23.5)	14 (26.9)
Transfer from another ICU, *n* (%)	0 (0)	1 (1.9)
Transfer from another hospital, (except from another ICU), *n* (%)	11 (21.6)	10 (19.2)
Operating Theatre/Recovery following EMERGENCY surgery, *n* (%)	11 (21.6)	7 (13.5)
Operating Theatre /Recovery following ELECTIVE surgery, *n* (%)	1 (2)	0 (0)
APACHE III Diagnosis^†‡^		
Respiratory, *n* (%)	25 (49)	19 (36.5)
Sepsis, *n* (%)	9 (17.6)	11 (21.2)
Gastrointestinal, *n* (%)	8 (15.7)	13 (25)
Cardiovascular, *n* (%)	2 (3.9)	3 (5.8)
Trauma, *n* (%)	4 (7.8)	4 (7.7)
Neurological, *n* (%)	1 (2)	0 (0)
Metabolic or endocrine disorder, *n* (%)	0 (0)	1 (1.9)
Renal, *n* (%)	0 (0)	0 (0)
Haematological, *n* (%)	1 (2)	0 (0)
Musculoskeletal or skin disorder, *n* (%)	1 (2)	0 (0)
Other, *n* (%)	0 (0)	1 (1.9)
RASS prior to randomisation, Median [IQR]**	− 3 [− 5 to − 2]	− 4 [− 5 to − 2]
Received dexmedetomidine prior to randomisation, *n* (%)	1 (2.1)	0 (0)
Received midazolam prior to randomisation, *n* (%)	16 (33.3)	17 (35.4)
Received propofol prior to randomisation, *n* (%)	32 (66.7)	35 (72.9)

*APACHE* Acute Physiology and Chronic Health Evaluation, *RASS* Richmond Agitation and Sedation Scale

Plus-minus values are ± SD. Listed are available baseline data for patients in the two groups who did not withdraw consent. There were no significant differences in baseline characteristics between the trial groups except for age (*p* = 0.04). Percentages may not total 100 because of rounding. ICU denotes intensive care unit, and IQR interquartile range

^§^The APACHE II score is a prediction tool for death and measures severity of disease in the ICU; scores range from 0 to 71, with higher scores indicating a greater severity of illness

^†^Conditions are listed according to the major disease categories in the APACHE III diagnostic codes

^‡^Patients with suspected or proven sepsis at randomisation may have been assigned to other APACHE III diagnostic categories, such as pneumonia and respiratory disorder

**The Richmond Agitation and Sedation Scale (RASS) is a tool to assess depth of sedation on a scale of − 5 to + 4, with negative values denoting increased sedation and positive values denoting increased agitation

One hundred and eleven patients were recruited between January 2017 and February 2018. Fifty-six were randomised to early sedation with dexmedetomidine and 55 to standard care. Five patients in the dexmedetomidine group and three in the standard group withdrew consent to continue, leaving 103 patients in the final analysis (Additional file 1: Appendix Fig. S3).

Patient characteristics at baseline were similar. The mean (SD) age of patients was 61 (15.9) years in the dexmedetomidine group and 66.6 (10.5) years in the usual-care group. Mean (SD) APACHE II scores were 20.3 (7.91) and 21.5 (6.91) in the dexmedetomidine and usual-care groups, respectively. An admission diagnosis of sepsis was similar between groups with 33 patients (64.7%) in the dexmedetomidine group and 33 patients (63.5%) in the usual-care group.

### ***Sedation ***(Table 2)

**Table 2:** Sedative and Analgesic management

	Dexmedetomidine (*n* = 51)	Usual care (*n* = 52)	*p*
Received dexmedetomidine, *n* (%)^§^	49 (96.1)	2 (3.8)	< 0.01
Received midazolam, *n* (%)	17 (33.3)	30 (57.7)	0.01
Received propofol, *n* (%)	43 (84.3)	48 (92.3)	0.21
Received fentanyl, *n* (%)	50 (98)	49 (94.2)	0.32
Received morphine, *n* (%)	11 (21.6)	18 (34.6)	0.14
Duration of dexmedetomidine infusion (days)	4 [2–7]	–	–
Duration of midazolam infusion (days)	1 [1–4]	3 [2–4]	0.04
Duration of propofol infusion (days)	5 [2–8]	4 [2–8]	0.96
Duration of fentanyl infusion (days)	6 [3–9]	6 [3–9]	0.89
Duration of morphine infusion (days)	1 [1–2]	2.5 [2–7]	0.05
Median dexmedetomidine dose (mcg/kg/day)	9.01 [5.0–14.8]	–	–
Median midazolam dose (mg/kg/day)	0.14 [0.08–0.38]	0.46 [0.20–0.93]	0.01
Median propofol dose (mg/kg/day)	7.26 [4.01–15.1]	9.38 [6.24–14.9]	0.38
Median fentanyl dose (mcg/kg/day)	12.2 [8.29–15.8]	11.1 [7.5–15.2]	0.58
Median morphine dose (mg/kg/day),	0.42 [0.07–0.81]	0.71 [0.15–1.7]	0.31
Sedation Index during first 48 h (RASS)*	2.08 [1.5–3.0]	2.26 [1.52–3.56]	0.45
Sedation Index while ventilated (RASS)*	1.91 [1.36–2.43]	2 [1.35–2.35]	0.4
Duration of ventilation (days)	3.73 [1.89–7.73]	5.07 [2.43–10.4]	0.91

Data given as mean (SD), median [95% CI]* Sedation index is calculated as the sum of negative Richmond Agitation Sedation Scale measurements divided by the total number of assessments

^§^In the 2 patients from the usual care group who received the median [95%] duration of infusion was 2.5 days [2–3] and a dose of 8.09 [3.76–12.4] (mcg/kg/day)

The two groups received significantly different sedation regimens. In the intervention group 95% of patients received dexmedetomidine versus 3.8% in the control group (*p* < 0.01). A third of the intervention group received midazolam versus 57.7% in the control group (*p* = 0.01). The intervention group received a median [IQR] dose of midazolam of 0.14 [0.08–0.38] mg/kg/day versus 0.46 [0.20–0.93] mg/kg/day in the control group (*p* = 0.01). There was no difference in sedation level (RASS) between groups within the first 48 h, or during the period of mechanical ventilation.

### ***Blood-borne stress markers ***(Tables 3, 4 and Fig. 1)

**Table 3:** Physiological variables, recorded at the time of blood sampling

	Dexmedetomidine (*n* = 51)	Usual care (*n* = 52)	*p*
Heart rate (bpm)	84.73 [80.71–88.75]	89.38 [85.38–93.38]	0.11
MAP (mmHg)	80.67 [78.34–83.0]	78.49 [76.16–80.82]	0.20
Minute volume (L/min)	8.90 [8.29–9.51]	9.24 [8.65–9.83]	0.43
Respiratory rate (breaths/min)	18.31 [17.23–19.39]	18.61 [17.55–19.67]	0.70
Temperature (°C)	37.31 [37.11–37.51]	37.17 [36.97–37.37]	0.50

Data given as mean [95% confidence interval]

*MAP* mean arterial pressure

**Table 4:** Blood-borne stress markers

Analyte	Normal range	Dexmedetomidine (*n* = 51)	Usual care (*n* = 52)	*p*
*Stress hormones*				
ACTH (ng/L)*	10–50	17.1 [15.1–19.5]	18.1 [15.9–20.5]	0.56
Aldosterone (pmol/L)*	0–400 (supine)	71.8 [50.4–102.3]	59.7 [42.1–84.8]	0.46
Adrenaline (nmol/L)*^‡^	< 3.5	0.32 [0.26–0.4]	0.38 [0.31–0.48]	0.25
Noradrenaline (nmol/L)*^‡^	< 1	4.27 [3.12–5.85]	6.2 [4.6–8.5]	0.09
Total cortisol (mU/L)*	140–640	515 [409–648]	618 [491–776]	0.26
*Anabolic and catabolic hormones*				
GH (mU/L) n*	Post-suppression < 0.5 post-stimulation > 10	2.77 [2.06–3.71]	1.97 [1.48–2.64]	0.1
Insulin (mU/L)*	2–23	16.9 [13.4–21.4]	16.0 [12.6–20.2]	0.73
Leptin (ng/ml)*	3.7–11.1	21.6 [16.7–27.9]	25.9 [20.1–33.4]	0.31
Oestradiol (pmol/L)*	< 180 (< 100 post-menopausal)	119 [95.4–149.6]	113 [90.6–141.5]	0.73
Prolactin (mU/L)*	Male 56–278, Female 58–416	510 [423–615]	458 [381–551]	0.42
Testosterone (nmol/L)*	Male 9–35, Female 0.3–2.6	1.39 [1.14–1.71]	1.56 [1.28–1.91]	0.42
fT3 (pmol/L)	3.5–6	3.4 [3.3–3.6]	3.4 [3.2–3.6]	0.54
T4 (pmol/L)	7–17	11.8 [11.1–12.5]	11.6 [10.9–12.3]	0.64
TSH (mU/L)*	0.3–4.5	1.22 [0.95–1.56]	1.34 [1.05–1.72]	0.58
*Lipid metabolism*				
Beta Hydroxybutyrate (mmol/L)*	< 0.2	0.17 [0.14–0.21]	0.19 [0.16–0.23]	0.46
HDL cholesterol (mmol/L)*	> 1	0.43 [0.34–0.54]	0.49 [0.39–0.62]	0.43
Total cholesterol (mmol/L)	< 6	2.8 [2.5–3.1]	2.7 [2.4–3.0]	0.42
Triglycerides (mmol/L)*	< 1.5	1.64 [1.43–1.86]	1.52 [1.33–1.73]	0.42
*Cardiac injury/dysfunction*				
BNP (ng/L)*	< 100	266 [202–352]	240 [182–316]	0.6
Troponin I (ng/L)*	< 0.04	0.11 [0.07–0.17]	0.09 [0.06–0.14]	0.58
*Other*				
Creatinine (µmol/L)*	Male 60–110 Female 45–90	105.2 [90.9–122]	97.7 [84.4–113.1]	0.48
Urea (mmol/L)	2.9–8.2	11.6 [9.9–13.2]	10.3 [8.7–11.9]	0.27
Albumin (g/L)	35–50	23.9 [22.3–25.4]	24.5 [23.0–26.0]	0.56
ALP (U/L)*	30–110	88.1 [76.2–101.8]	81.3 [70.6–93.7]	0.43
ALT (U/L)*	< 45	49.3 [36.5–66.7]	41.7 [31–56]	0.43
AST (U/L)*	< 35	64.2 [47.2–87.1]	61.6 [45.9–83.2]	0.85
Glucose (nmol/L)	3–7.8	8.7 [8.1–9.2]	8.3 [7.8–8.9]	0.43
Lactate (mmol/L)*	0.5–2.2	1.36 [1.2–1.53]	1.23 [1.09–1.38]	0.25
pH	Male 7.32–7.43, Female 7.35–7.45	7.38 [7.37–7.40]	7.38 [7.36–7.39]	0.68
SBE (mmol/L)	− 2—> 3	0.69 [− 0.61–1.99]	0.9 [− 0.38–2.17]	0.82
Platelets (× 10^9^/L)	140–400	221 [197–246]	205 [181–229]	0.33
PT (secs)	9–13	14.9 [13.5–16.2]	15.1 [13.8–16.3]	0.83
WCC (× 10^9^/L)	3.5–11	14.3 [12.6–16.0]	12.6 [10.9–14.2]	0.14

*ACTH* adrenocorticotropic hormone, *ALP* alkaline phosphatase, *ALT* alanine transferase, *AST* aspartate transferase, *BNP* brain-natriuretic peptide, *FT3* free triiodothyronine, *GH* growth hormone, *HDL* high density lipoprotein, *PT* prothrombin time, *SBE* standard base excess, *T4* thyroxine, *TSH* thyroid stimulating hormone, *WCC* white cell count

*p* value represents the global comparison of DEX versus Usual Care derived from Analysis of variance

Data given as mean [95% CI] except where indicated

*Geometric mean

^‡^No data on exogenous administration of indicated biomarkers was available

**Fig. 1:**
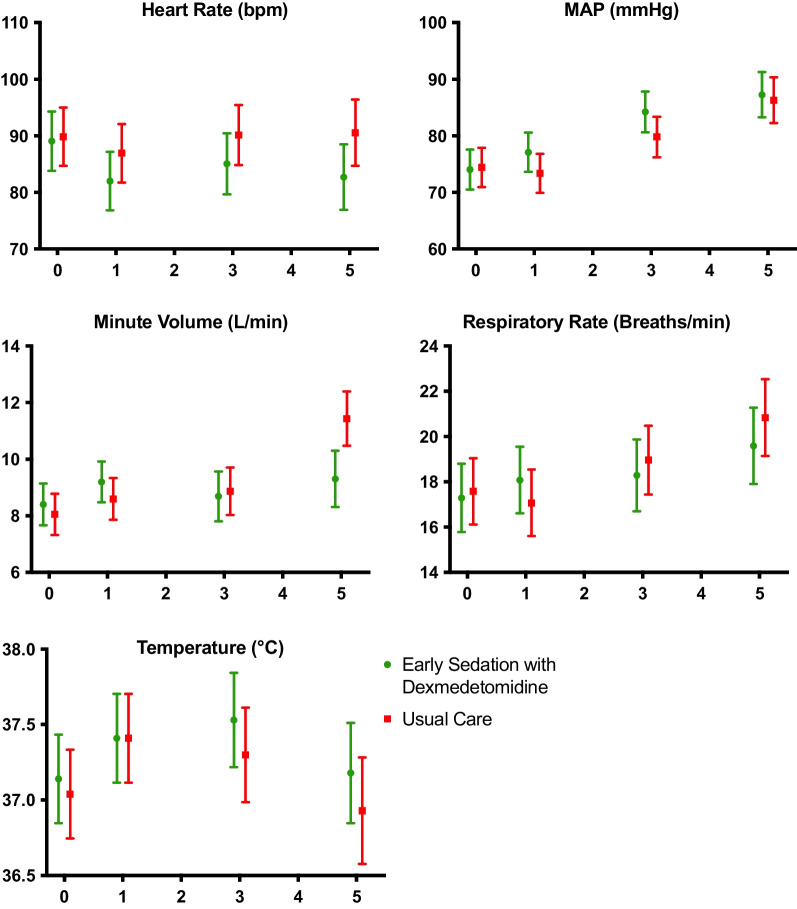
Physiological variables over time. x-axes denote days post-randomisation. All data are presented as mean [95%CI]

Overall comparison of results from all timepoints for all blood-borne markers of stress and physiological variables of heart rate, mean arterial pressure, respiratory rate, minute volume and temperature showed no significant differences between the dexmedetomidine and usual-care groups. There were no significant differences between groups at individual timepoints nor in longitudinal variance over time for any analyses (Additional file 1: Appendix Figs. S4 and S5). Compliance with the protocol was high with 98.2% (*n* = 54) in the dexmedetomidine group having blood samples taken at baseline, versus 97.1% (*n* = 48) in the usual care group (*p* = 0.27). By the end of the study period 69.8% (*n* = 30) in the dexmedetomidine group had blood samples taken, versus 72.8% (*n* = 25) in the usual care group (*p* = 0.48) (Additional file 1: Appendix Table S6, Fig. S5).

Post hoc analysis of the 59 patients in the older age group (age above > 63.7 years), and in the 63 patients admitted with a diagnosis of sepsis, showed no significant differences in any physiological or biochemical marker between the dexmedetomidine and usual-care groups (Additional file 1: Appendix Tables S2–S5).

### Co-administration of medications

At baseline, 9.8% (*n* = 5) patients in the usual care group received angiotensin 2 inhibitors versus 26.9% (*n* = 14) in the dexmedetomidine group at baseline (*p* = 0.03). There was no significant difference at baseline in prescription rates for beta blockers, ACE inhibitors, steroids or TPN (Additional file 1: Appendix Tables S7, S8 and Fig. S6). On day 3, 31% (*n* = 16/51) of the dexmedetomidine group received steroid versus 14.7% (7/51) in the usual care group (*p* = 0.033). There was no difference in the rate of steroid prescription at baseline, day 3 or day 5.

### ***Outcomes ***(Table 5)

**Table 5:** Patient outcomes

	Dexmedetomidine (*n* = 51)	Usual care (*n* = 52)	*p*
Hospital length of stay (days)	19.7 [9.85–33.4]	18.8 [11.6–32]	0.98
ICU length of stay (days)	6.4 [3.98–11.9]	7.85 [4–15]	0.75
Duration of ventilation (days)	3.73 [1.89–9.32]	5.07 [2.45–10.4]	0.53
Died before ICU discharge, *n* (%)	6 (11.8)	8 (15.4)	0.59
Died before hospital discharge, *n* (%)	9 (17.7)	10 (19.2)	0.76
Died before 90 days, *n* (%)	9 (17.7)	10 (19.2)	0.76
Died before 180 days, *n* (%)	10 (19.6)	11 (21.2)	0.81

Data given as median [IQR]

There were no significant differences in between-group ICU or hospital length of stay, duration of ventilation or mortality up to 180 days.

## Discussion and conclusions

In two groups treated with either dexmedetomidine or usual care targeting light sedation, we found no differences in physiologically or blood-borne biomarker of the stress response. There was also no significant difference in downstream organ function.

While our study was not able to detect a difference, it remains possible that the type of sedative drug choice resulted in different stress responses that were not detected due to the relatively small sample size in our study. However, even if present, the clinical relevance of any such difference would be questionable in the context of the lack of primary outcome difference in the main SPICE-III trial. The main study demonstrated that, among older patients, a lower proportion died in the dexmedetomidine arm of the study compared to those treated with usual care, while the converse was true for younger patients. Reasons for this are not clear but might be explained by differences in rates of dexmedetomidine use varying according to age. In our sub-study, we were unable to identify any significant effect on outcomes, nor in components of the stress response in the older population while acknowledging the relatively small sample size.

Several studies have shown beneficial clinical outcomes with dexmedetomidine [16–18]. Its use has been reported to increase albumin levels [19] and reduce inflammation, as represented by C-reactive protein and procalcitonin, which also serve as biomarkers of the stress response. Our study was weakened by omitting the testing of CRP and procalcitonin, their inclusion would have permitted a broader examination of stress response. Ohta et al. demonstrated reductions in both CRP and procalcitonin with dexmedetomidine therapy in mechanically ventilated patients with sepsis. These differences were small but do lend credence to the concept of off-axis effects of different sedative agents on the output of the stress system. In contrast, our study showed no significant differences with sedation therapy in any domain that represents either the stress response, the inflammatory response or the acute phase reactant response in either the whole population or the septic sub-group.

Our study has several strengths. It utilised four separate centres participating in a large multi-centre randomised controlled trial and took blood samples from over 100 patients at four separate time points with marginal loss of participants due to withdrawal of consent. This sub-study comprised a broad population of critically ill patients, there was excellent compliance with the study sedation protocol, and early achievement of light levels of sedation in both arms. A few differences were seen with this sub-population as compared to the main SPICE-III studies. The proportion of patients admitted with a cardiovascular diagnosis was 4.9% in this study as compared to 14.8% in the SPICE-III trial. The sedative proportions and doses given in this study were similar to the SPICE-III study, except a higher proportion in the sub-study achieved light sedation levels (RASS − 2 to + 1) within the first 48 h. The illness severity in our population was significant, as evidenced by the high APACHE-II scores and patient outcomes, which were comparable to SPICE-III. Small patient numbers limited our study. While adequately powered to detect an approximate change of 20% for each marker, a difference of this magnitude may not represent a difference that is of pathophysiological relevance. Moreover, while a smaller *p* value of 0.01 was used to negate the chance of a type-I error, there were ultimately insufficient patients to fully account for the number of comparisons presented.

Our study was limited by the absence of data on the administered doses of insulin, epinephrine, norepinephrine and thyroxine. This reduces the ability to interpret the corresponding measured hormone levels or downstream physiological variables. There is the small possibility that unmeasured differences in the administration of drugs between groups might mask real differences in the measured outcomes. We believe that the difference in steroid prescription rates seen on day 1 would have had a negligible effect upon the results, particularly as the rate of steroid prescription was low in both groups and the difference was not sustained through the study period.

The dose of dexmedetomidine given in the dexmedetomidine group overall was equivalent 0.38 mcg/kg/h. While low, this dose is the average for the whole duration of the infusions from commencement to weaning. When considered alongside the significant use of midazolam and propofol in both groups, it is possible the difference in sedation management was not profound enough to yield a measurable difference in stress response. The lack of difference in the stress markers seen between sedation strategies may have been impacted by the timing of the blood tests. As a pragmatic decision, each patient had their blood sampled as close to the time of intubation as possible, subsequent blood draws were performed on the morning of the respective day post-intubation due to practical concerns. This led to a non-standardised time interval following intubation for subsequent blood draws. As there was no significant difference in time interval between treatment groups at each time point, it is unlikely that this influenced the outcome (Additional file 1: Appendix Fig. S7); however, our data limitations preclude meaningful inference pertaining to causality. Many of the blood-borne markers demonstrated predictable variation over time; it is possible that sedation choice might modify these patterns, but the non-uniform time intervals may have masked this effect. However, we consider the likelihood of any clinically significant difference is negligible.

In conclusion, early sedation with dexmedetomidine in ventilated critically ill adults resulted in no difference in physiological or blood-borne biomarkers of stress as compared to usual-care sedation. Due to the scope and limitations of this data we are unable to infer any potential advantage in using either sedation strategy.

## Supplementary Information

**Additional file 1.** **Appendix 1.** Supplementary figures and tables.

## Data Availability

The data that support the findings of this study are available from the corresponding author upon reasonable request.

## Abbreviations

ACE: Angiotensin converting enzyme
ACTH: Adrenocorticotropic hormone
ALP: Alkaline phosphatase
ALT: Alanine transferase
ANZCTR: Australian New Zealand clinical trials registry
APACHE: Acute physiology and chronic health evaluation
AST: Aspartate transferase
BNP: Brain natriuretic peptide
CRP: C-reactive protein
FT3: Free triiodothyronine
GABA: Gamma-aminobutyric acid
GH: Growth hormone
HDL: High density lipoprotein
MAP: Mean arterial pressure
PT: Prothrombin time
RASS: Richmond agitation-sedation scale
SBE: Standard base excess
SPICE: Sedation practices in intensive care evaluation
T4: Thyroxine
TPN: Total parenteral nutrition
TSH: Thyroid stimulating hormone
WCC: White cell count
