# Supplementary material for: Stress response during early sedation with dexmedetomidine compared with usual-care in ventilated critically ill patients
Source: Crit Care. 2022 Nov 22;26:359. doi: 10.1186/s13054-022-04237-0 (PMC9682690; doi:10.1186/s13054-022-04237-0)
Supplement: Supplementary file 1 — Additional file 1. Appendix 1. Supplementary figures and tables. [file 13054_2022_4237_MOESM1_ESM.docx]

Appendix 1

Figure S1. Dexmedetomidine Algorithm

- Medical Schools

**Until sedation is no longer required or day 28**

**≤ -3**

**Inadequate**

**Titrate**

**0-1 µg/kg/h**

**RASS**

**-2 - +1**

**Add propofol**

**10-70 mg/h**

**-2 to +1**

**↓ Dexmed by**

**0.2 µg/kg/h**

**Titrate**

**0-70 mg/h**

**Propofol can be increased to 200 mg/h**

**Reduce propofol first when titrating down**

**Opiates given via bolus/infusion as needed**

**Dexmedetomidine**

**RASS**

Figure S2. Standard Sedation Algorithm

**Until sedation is no longer required or day 28**

**≤ -3**

**Inadequate**

**RASS**

**-2 - +1**

**Clinician’s directed sedative titration**

**Propofol / midazolam / others**

**-2 to +1**

**Propofol/midazolam/others bolus/infusion**

**Opiates given via bolus/infusion as needed**

**Dexmedetomidine is precluded**

**RASS**

**Usual care**

Table S1

**SPICE-III Inclusion Criteria**

- Subject has been intubated and is receiving mechanical ventilation
- The treating clinician expects that the patient will remain intubated until the day after tomorrow (unlikely to be extubated the following day).
- The patient requires immediate ongoing sedative medication for comfort, safety, and to facilitate the delivery of life support measures.

**SPICE-III Exclusion Criteria**

- Age less than 18 years
- Patient is pregnant and/or lactating
- Has been intubated (excluding time spent intubated within an operating theatre or transport) for greater than 12 hours in an intensive care unit
- Proven or suspected acute primary brain lesion such as traumatic brain injury, intracranial haemorrhage, stroke, or hypoxic brain injury.
- Proven or suspected spinal cord injury or other pathology that may result in permanent or prolonged weakness
- Admission as a consequence of a suspected or proven drug overdose or burns.
- Administration of ongoing neuromuscular blockade
- Mean arterial blood (MAP) pressure that is less than 50 mmHg despite adequate resuscitation and vasopressor therapy at time of randomisation
- Heart rate less than 55 beats per minute unless the patient is being treated with a beta blocker or a high grade atrio-ventricular block in the absence of a functioning pacemaker
- Known sensitivity to any of the study medications or the constituents of propofol (egg, soya or peanut protein
- Acute fulminant hepatic failure
- Patient has been receiving full time residential nursing care.
- Death is deemed to be imminent or inevitable during this admission and either the attending physician, patient or substitute decision maker is not committed to active treatment.
- Patient has an underlying disease that makes survival to 90 days unlikely
- Patient has been previously enrolled in the SPICE-III study.

**Figure S3 Consort Diagram**

Analysed (n=51)

Discontinued intervention

(withdrew consent) (n= 5)

Allocated to intervention (n=56)

♦ Received allocated intervention (n=56 )

Discontinued intervention

(withdrew consent) (n=3)

Allocated to intervention (n=55)

♦ Received allocated intervention (n=55 )

Analysed (n=52)

## Allocation

## Analysis

## Follow-Up

Randomized (n=111)

**Figure S4 - Stress panel results over time**

**
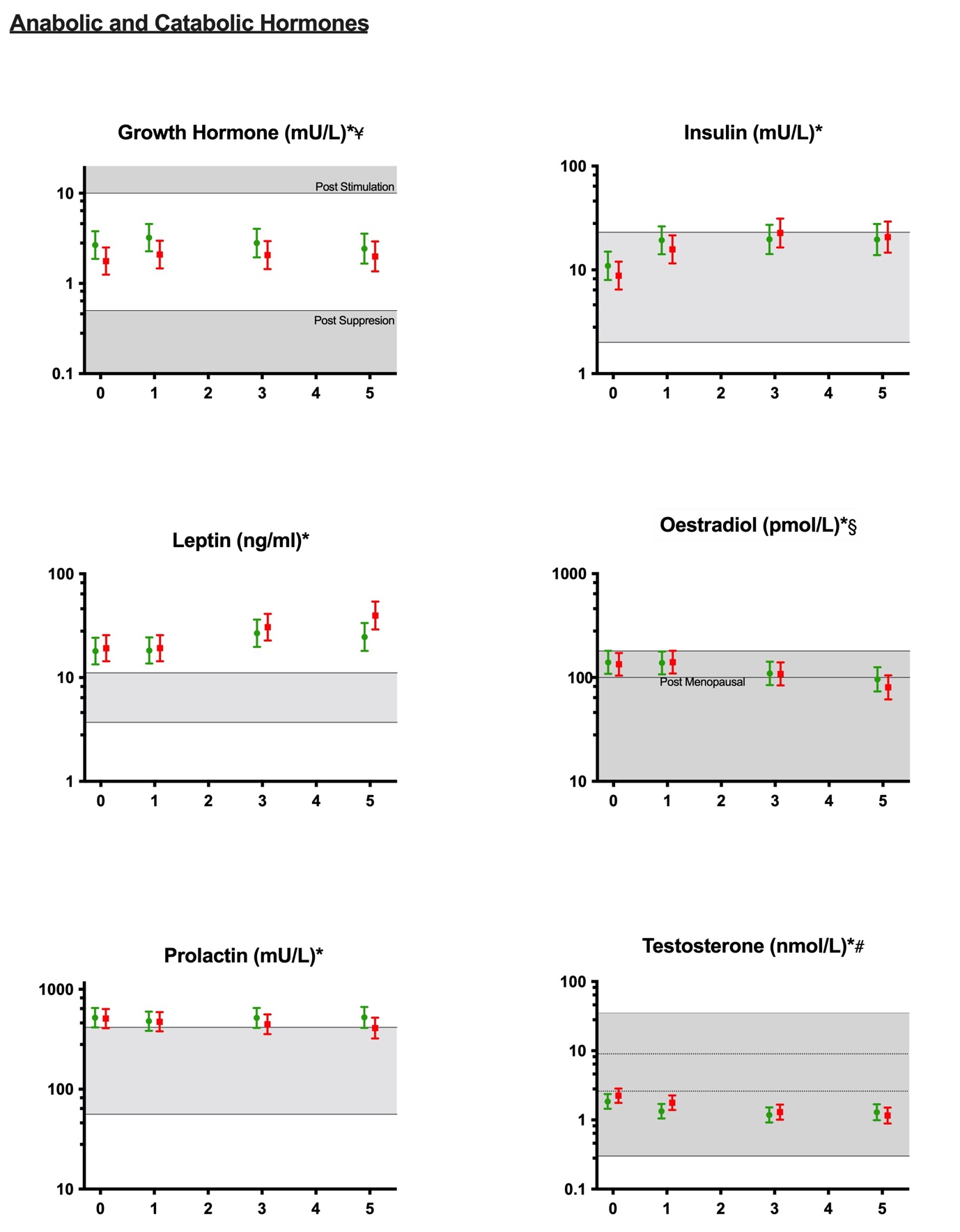

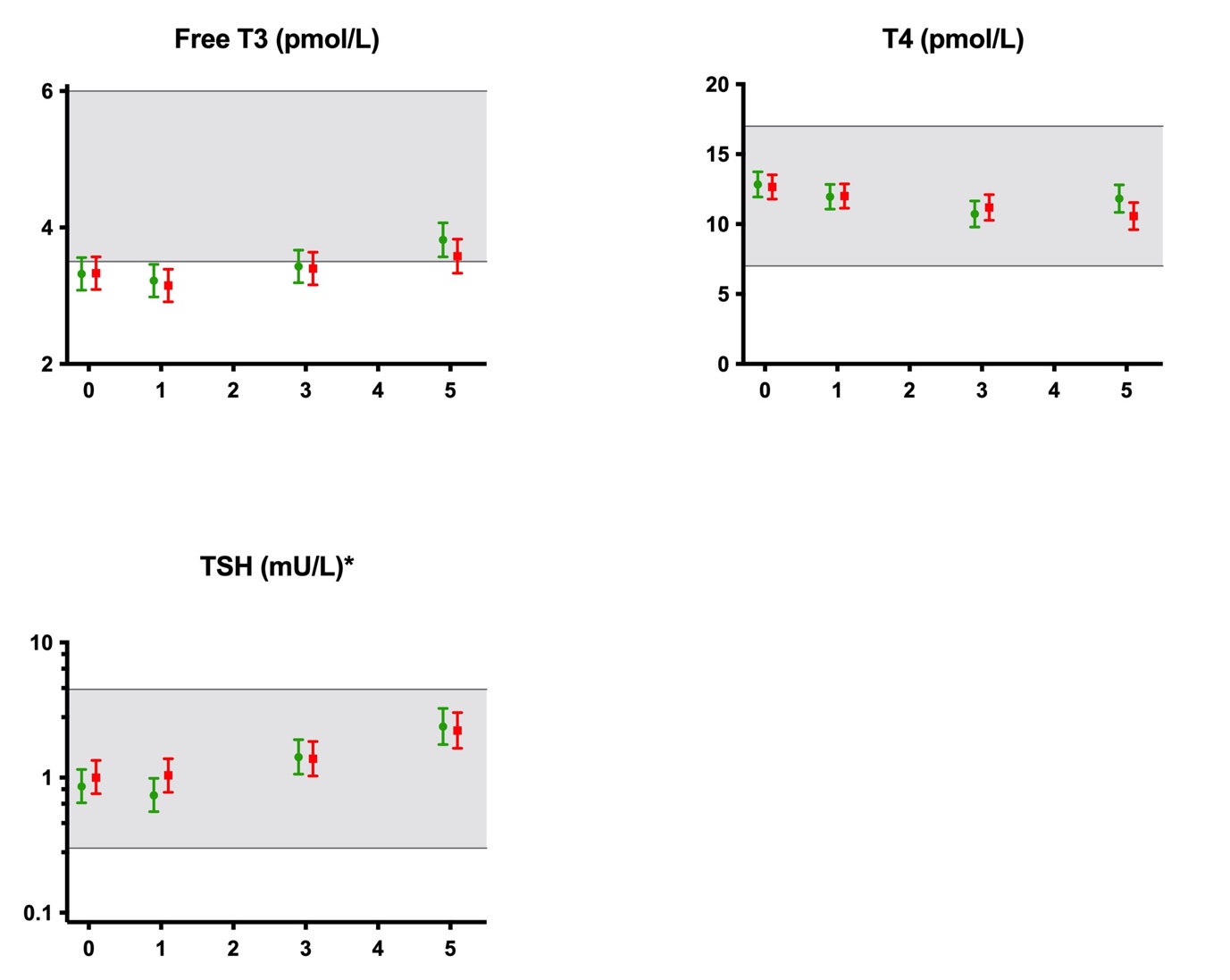
**

**
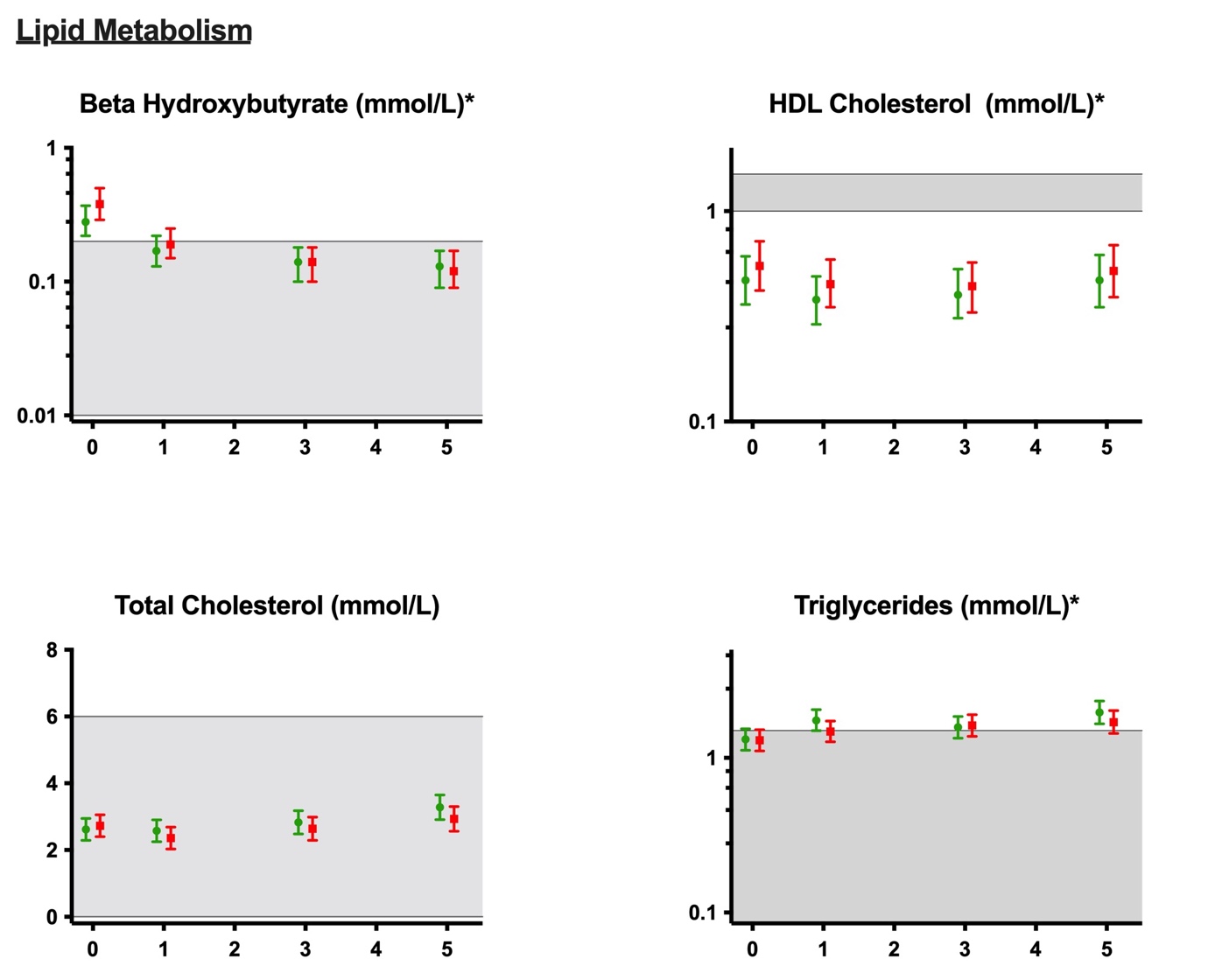
**

**
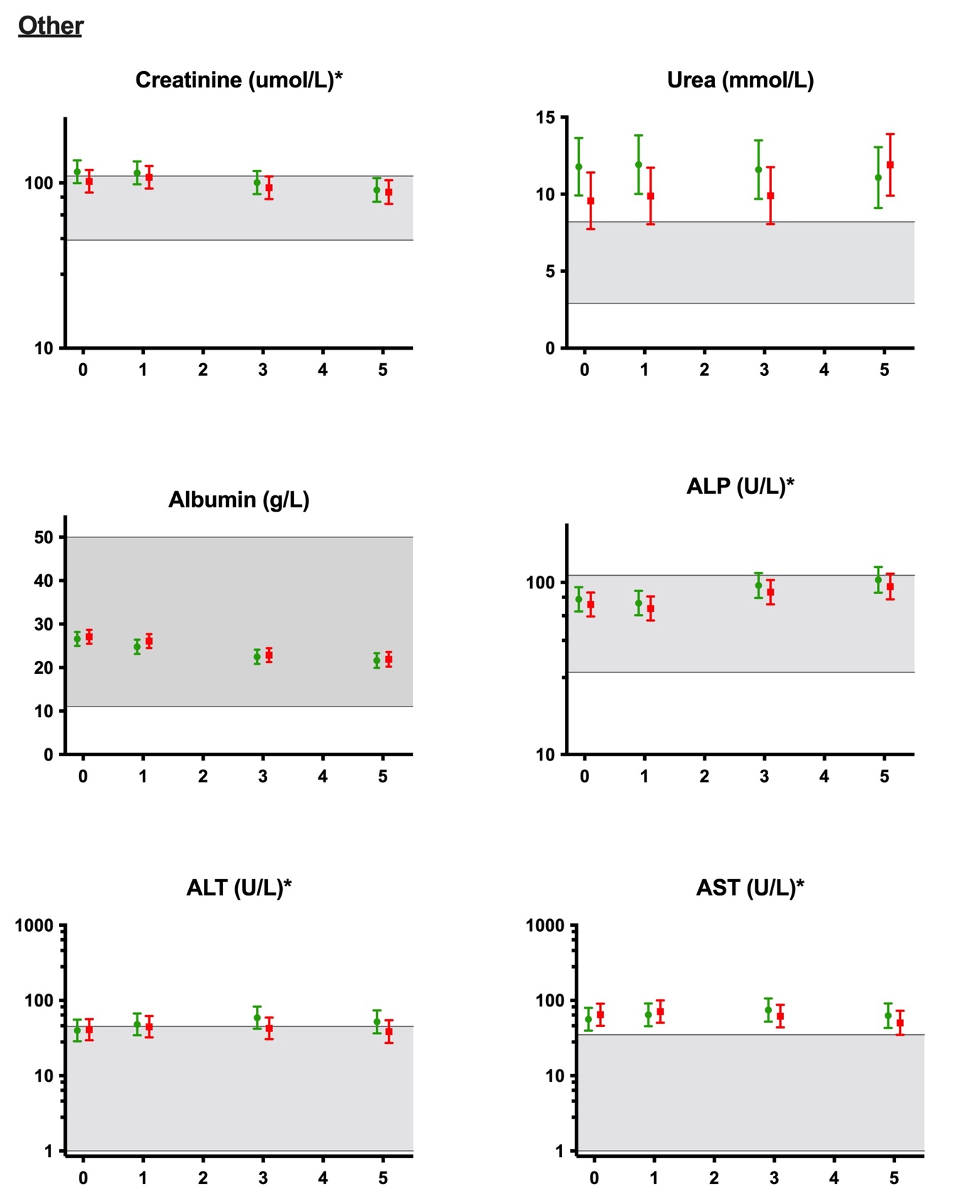

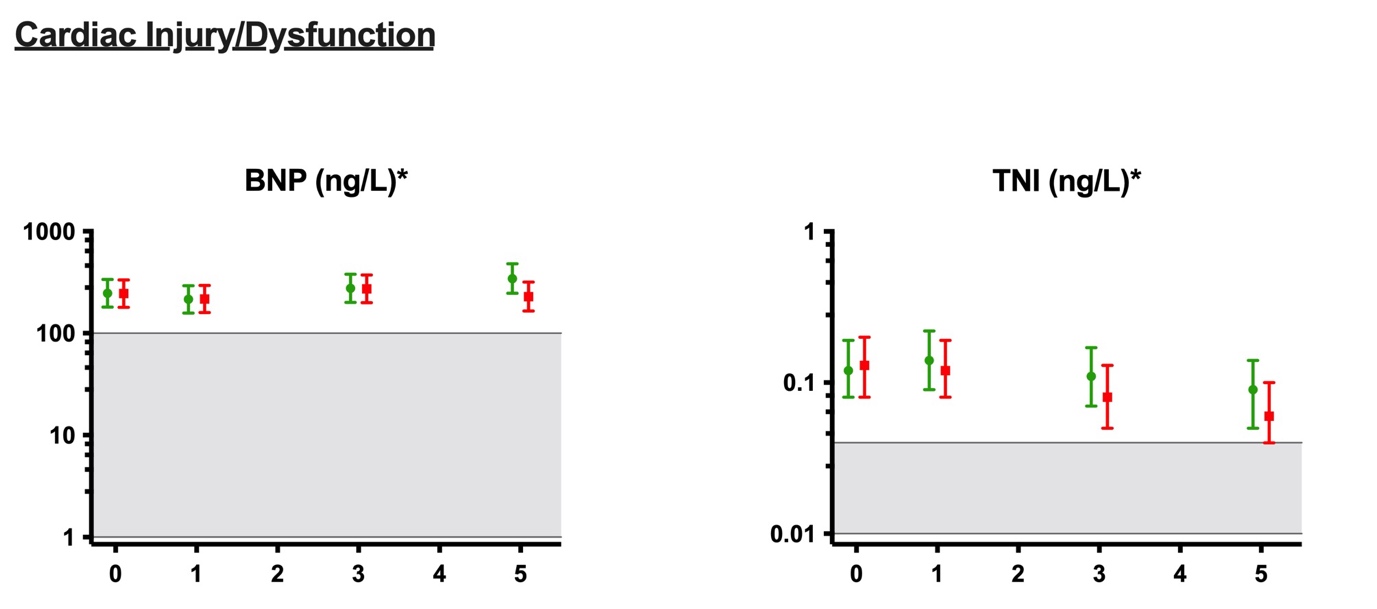

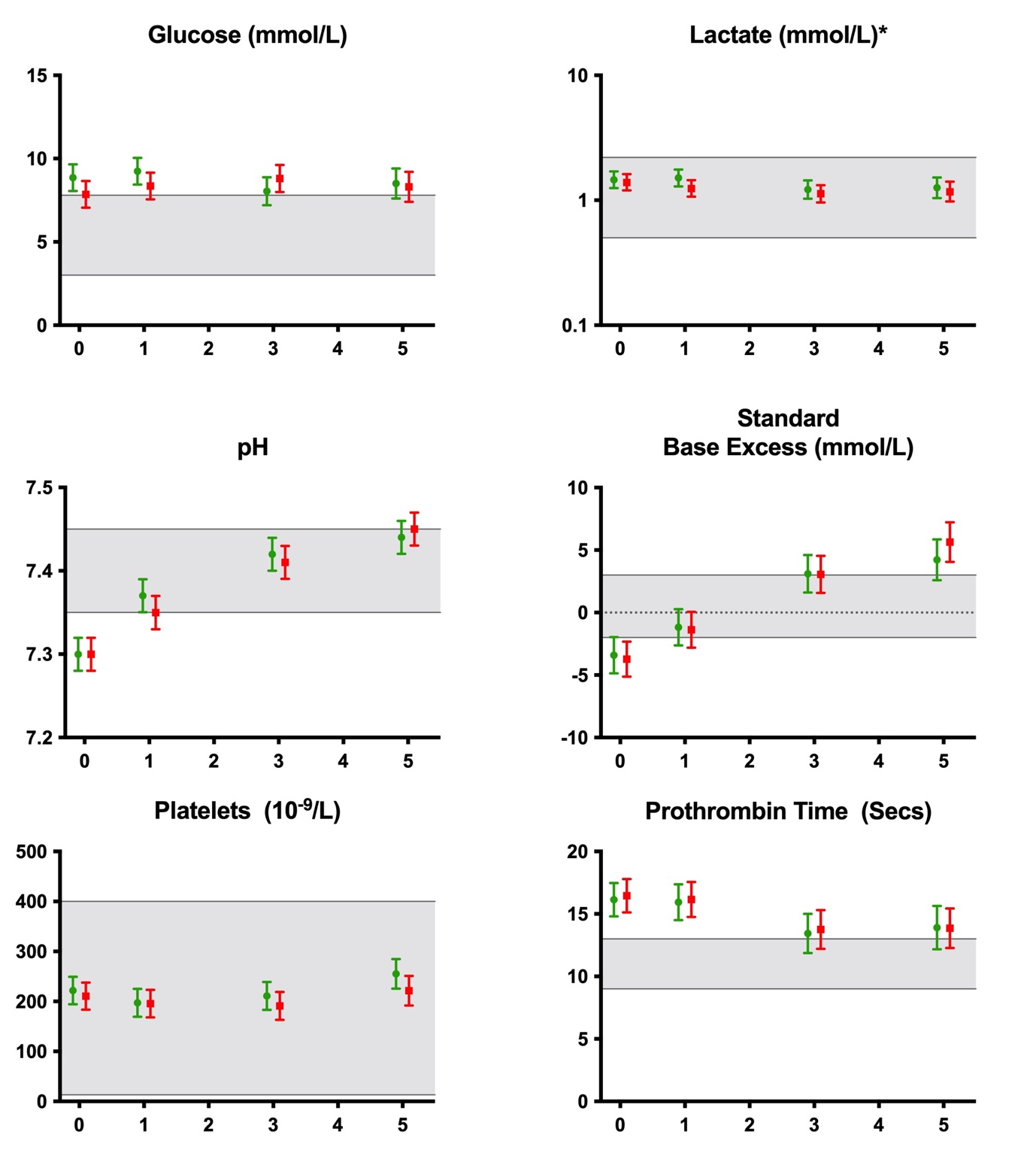

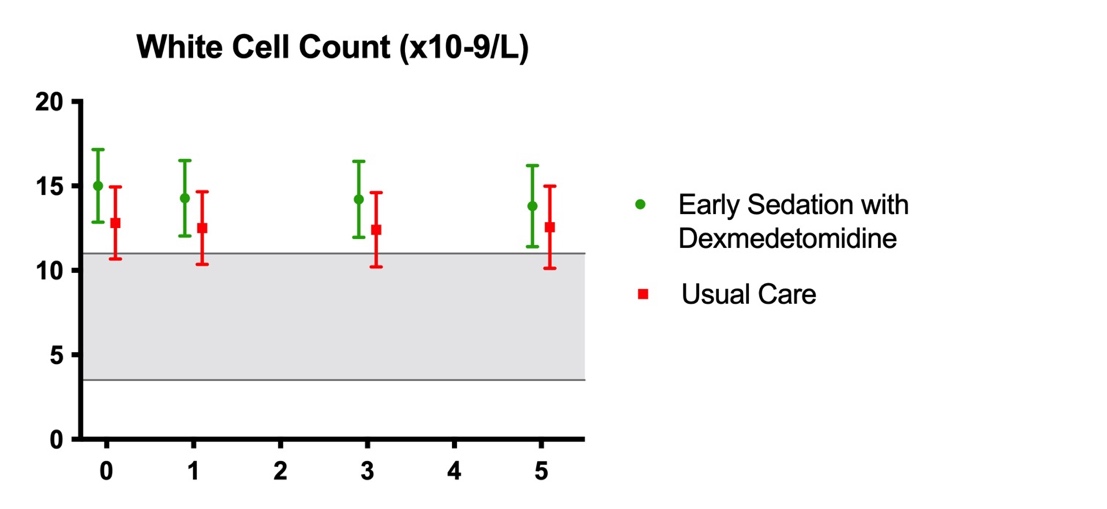
**

X-axis denotes days post randomisation

*geometric means for log normally distributed data, otherwise all data are presented as mean [95%CI]

Shaded areas represent normal ranges or reference levels.

†Total Cortisol reference range: 0800hrs 140-640 mU/L, 1600hrs 80-440mU/L, 2400hrs <330 mU/L

¥Growth Hormone reference ranges: post-suppression normal <0.5mU/L, post-stimulation normal >10mU/L

§Oestradiol reference range: normal <180pmol/L, post-menopausal normal <100pmol/L

#Testosterone reference ranges: Male 9-35nmol/L, Female 0.3-2.6nmol/L

**Table S2 – Physiological variables in patients aged >63.7yrs**

|  | **Dexmedetomidine (n=27)** | **Usual Care**  **(n=33)** | **p** |
| --- | --- | --- | --- |
| Heart rate (bpm) | 84.7 [80.6-160.07] | 89.4 [85.34-169.34] | 0.12 |
| MAP (mmHg) | 80.48 [78.1-154.29] | 78.67 [76.31-150.76] | 0.30 |
| Minute volume (L/min) | 8.88 [8.27-16.53] | 9.25 [8.66-17.27] | 0.40 |
| Respiratory rate (breaths/min) | 18.27 [17.18-34.23] | 18.65 [17.58-35] | 0.63 |
| Temperature (°C) | 36.76 [36.07-71.05] | 37.24 [36.55-71.99] | 0.35 |

Abbreviations: MAP mean arterial pressure

Data given as mean [95% confidence interval]

**Table S3 – Stress panel results in patients aged >63.7yrs**

| **Analyte** | | **Normal Range** | **Dexmedetomidine**  **(n=27)** | **Usual Care**  **(n=33)** | | **p** |
| --- | --- | --- | --- | --- | --- | --- |
| Stress Hormones |  |  |  | |  | |
| ACTH (ng/L) * | | 10-50 | 16.74 [14.06-19.93] | 19.24 [16.45-22.51] | | 0.24 |
| Aldosterone (pmol/L) * | | 0-400 (supine) | 57.67 [34.81-95.55] | 52.13 [33.05-82.24] | | 0.77 |
| Adrenaline (nmol/L) * | | <3.5 | 0.38 [0.28-0.52] | 0.41 [0.31-0.55] | | 0.68 |
| Noradrenaline (nmol/L) * | | <1 | 5.7 [4.03-8.05] | 8.01 [5.86-10.95] | | 0.14 |
| Total cortisol (mU/L) * | | 140-640 | 746 [587-947] | 628 [506-778] | | 0.28 |
| Anabolic and Catabolic Hormones |  |  |  | |  | |
| GH (mU/L) n* | | post-suppression <0.5 post-stimulation >10 | 2.51 [1.76-3.58] | 1.61 [1.17-2.22] | | 0.07 |
| Insulin (mU/L) * | | 2-23 | 15.9 [11.55-21.88] | 17.69 [13.26-23.6] | | 0.62 |
| Leptin (ng/ml) * | | 3.7-11.1 | 24.3 [17.96-32.87] | 32.44 [24.72-42.58] | | 0.16 |
| Oestradiol (pmol/L) * | | <180 (<100 post-menopausal) | 112 [81-154] | 116 [87-154] | | 0.88 |
| Prolactin (mU/L) * | | Male 56-278, Female 58-416 | 633 [494-811] | 485 [387-606] | | 0.11 |
| Testosterone (nmol/L) * | | Male 9-35, Female 0.3-2.6 | 1.45 [1.1-1.9] | 1.37 [1.07-1.75] | | 0.75 |
| fT3 (pmol/L) | | 3.5-6 | 3.32 [3.09-6.18] | 3.26 [3.06-6.1] | | 0.71 |
| fT4 (pmol/L) | | 7-17 | 11.5 [10.64-21.29] | 12.01 [11.24-22.42] | | 0.38 |
| TSH (mU/L) * | | 0.3-4.5 | 1.42 [1.02-1.99] | 1.32 [0.97-1.79] | | 0.74 |
| Lipid Metabolism |  |  |  | |  | |
| Beta Hydroxybutyrate (mmol/L) * | | <0.2 | 0.21 [0.16-0.28] | 0.21 [0.16-0.26] | | 0.81 |
| HDL cholesterol (mmol/L) * | | >1 | 0.53 [0.39-0.71] | 0.49 [0.37-0.63] | | 0.67 |
| Total cholesterol (mmol/L) | | <6 | 2.91 [2.54-5.17] | 2.52 [2.19-4.46] | | 0.13 |
| Triglycerides (mmol/L) * | | <1.5 | 1.66 [1.4-1.95] | 1.42 [1.22-1.64] | | 0.16 |
| Cardiac injury/dysfunction |  |  |  | |  | |
| BNP (ng/L) * | | <100 | 340 [229-507] | 273 [190-391] | | 0.41 |
| Troponin I (ng/L) * | | <0.04 | 0.14 [0.08-0.25] | 0.11 [0.06-0.18] | | 0.44 |
| Other |  |  |  | |  | |
| Creatinine (µmol/L) * | | Male 60-110 Female 45-90 | 127 [104-155] | 104.74 [87.67-125.13] | | 0.15 |
| Urea (mmol/L) | | 2.9 - 8.2 | 12.86 [10.98-22.49] | 11.28 [9.58-19.65] | | 0.22 |
| Albumin (g/L) | | 35-50 | 23.98 [21.97-44.09] | 23.72 [21.9-43.86] | | 0.85 |
| ALP (U/L) * | | 30-110 | 86.19 [70.75-104.99] | 86.54 [72.55-103.22] | | 0.98 |
| ALT (U/L) * | | <45 | 45.6 [32.56-63.85] | 33.49 [24.77-45.26] | | 0.17 |
| AST (U/L) * | | <35 | 58.87 [41.62-83.27] | 52.81 [38.69-72.07] | | 0.64 |
| Glucose (nmol/L) | | 3-7.8 | 8.69 [8.03-16.07] | 8.4 [7.8-15.6] | | 0.52 |
| Lactate (mmol/L) * | | 0.5-2.2 | 1.29 [1.11-1.5] | 1.22 [1.07-1.4] | | 0.58 |
| pH | | Male 7.32-7.43, Female 7.35-7.45 | 7.38 [7.35-14.43] | 7.38 [7.36-14.43] | | 0.90 |
| SBE (mmol/L) | | -2 -> 3 | 0.55 [-1.1--1.31] | 0.99 [-0.49--0.21] | | 0.70 |
| Platelets (x10^9^/L) | | 140 - 400 | 231.99 [203.83-413.88] | 211 [186-377] | | 0.29 |
| PT (secs) | | 9-13 | 14.62 [13.3-26.74] | 14.83 [13.67-27.39] | | 0.82 |
| WCC (x10^9^/L) | | 3.5-11 | 13.52 [11.52-23.6] | 12.82 [11.03-22.53] | | 0.61 |

Abbreviations: ACTH adrenocorticotropic hormone, ALP alkaline phosphatase, ALT alanine transferase, AST aspartate transferase, BNP brain-natriuretic peptide, FT3 free triiodothyronine, GH growth hormone, HDL high density lipoprotein, PT prothrombin time, SBE standard base excess, T4 thyroxine, TSH thyroid stimulating hormone, WCC white cell count.

Data given as mean [95% CI] except where indicated

*Geometric mean

**Table S4 Physiological Variables in patients identified as having Sepsis**

|  | **Dexmedetomidine (n=33)** | **Usual Care**  **(n=32)** | **p** |
| --- | --- | --- | --- |
|  |  |  |  |
| Heart rate (bpm) | 84.4 [79.45-158.25] | 92.7 [87.79-174.57] | 0.02 |
| MAP (mmHg) | 80.08 [77.41-153.09] | 77.17 [74.54-147.45] | 0.13 |
| Minute volume (L/min) | 8.91 [8.12-16.31] | 9.92 [9.17-18.36] | 0.07 |
| Respiratory rate (breaths/min) | 19.41 [18.03-36.04] | 19.6 [18.23-36.43] | 0.86 |
| Temperature (°C) | 37.23 [36.96-72.58] | 37.16 [36.91-72.47] | 0.75 |

Abbreviations: MAP, mean arterial pressure

Data given as mean [95% confidence interval]

**Table S5 – Stress panel results in patients identified as having Sepsis**

| **Analyte** | | **Normal Range** | **Dexmedetomidine**  **(n=33)** | **Usual Care**  **(n=32)** | **p** |
| --- | --- | --- | --- | --- | --- |
| Stress Hormones |  |  |  |  | |
| ACTH (ng/L) * | | 10-50 | 18.18 [15.45-21.39] | 17.96 [15.29-21.1] | 0.91 |
| Aldosterone (pmol/L) * | | 0-400 (supine) | 66.75 [42.95-103.75] | 46.9 [30.24-72.72] | 0.26 |
| Adrenaline (nmol/L) * | | <3.5 | 0.31 [0.24-0.4] | 0.36 [0.28-0.47] | 0.41 |
| Noradrenaline (nmol/L) * | | <1 | 4.19 [2.85-6.15] | 7.71 [5.26-11.31] | 0.03 |
| Total cortisol (mU/L) * | | 140-640 | 539 [413-703] | 688 [528-896] | 0.20 |
| Anabolic and Catabolic Hormones |  |  |  |  | |
| GH (mU/L) n* | | post-suppression <0.5 post-stimulation >10 | 3.33 [2.24-4.93] | 2.17 [1.46-3.21] | 0.13 |
| Insulin (mU/L) * | | 2-23 | 16.83 [12.28-23.05] | 19.47 [14.24-26.61] | 0.51 |
| Leptin (ng/ml) * | | 3.7-11.1 | 18.59 [13.37-25.85] | 31.96 [23.02-44.37] | 0.02 |
| Oestradiol (pmol/L) * | | <180 (<100 post-menopausal) | 126 [94-170] | 127 [95-171] | 0.96 |
| Prolactin (mU/L) * | | Male 56-278, Female 58-416 | 544 [423-700] | 521 [405-670] | 0.81 |
| Testosterone (nmol/L) * | | Male 9-35, Female 0.3-2.6 | 1.47 [1.14-1.9] | 1.34 [1.04-1.74] | 0.63 |
| fT3 (pmol/L) | | 3.5-6 | 3.44 [3.19-6.38] | 3.33 [3.08-6.17] | 0.56 |
| fT4 (pmol/L) | | 7-17 | 11.18 [10.41-20.8] | 11.3 [10.54-21.04] | 0.82 |
| TSH (mU/L) * | | 0.3-4.5 | 1.39 [1.01-1.9] | 1.23 [0.9-1.69] | 0.60 |
| Lipid Metabolism |  |  |  |  | |
| Beta Hydroxybutyrate (mmol/L) * | | <0.2 | 0.17 [0.13-0.22] | 0.18 [0.15-0.23] | 0.67 |
| HDL cholesterol (mmol/L) * | | >1 | 0.35 [0.26-0.48] | 0.39 [0.29-0.52] | 0.66 |
| Total cholesterol (mmol/L) | | <6 | 2.75 [2.41-4.89] | 2.39 [2.05-4.18] | 0.14 |
| Triglycerides (mmol/L) * | | <1.5 | 1.78 [1.51-2.1] | 1.61 [1.36-1.9] | 0.38 |
| Cardiac injury/dysfunction |  |  |  |  | |
| BNP (ng/L) * | | <100 | 315 [223-445] | 258 [182-364] | 0.41 |
| Troponin I (ng/L) * | | <0.04 | 0.1 [0.06-0.15] | 0.08 [0.05-0.12] | 0.50 |
| Other |  |  |  |  | |
| Creatinine (µmol/L) * | | Male 60-110 Female 45-90 | 110 [91-133] | 109.34 [90.44-132.21] | 0.95 |
| Urea (mmol/L) | | 2.9 - 8.2 | 11.64 [9.75-20.07] | 11.25 [9.38-19.33] | 0.77 |
| Albumin (g/L) | | 35-50 | 23.15 [21.33-42.73] | 23.58 [21.77-43.6] | 0.74 |
| ALP (U/L) * | | 30-110 | 93.09 [77.45-111.9] | 95.47 [79.71-114.35] | 0.85 |
| ALT (U/L) * | | <45 | 46.03 [30.43-69.63] | 44.68 [29.76-67.09] | 0.92 |
| AST (U/L) * | | <35 | 59.74 [39.83-89.6] | 66.39 [44.62-98.79] | 0.71 |
| Glucose (nmol/L) | | 3-7.8 | 8.87 [8.11-16.29] | 9.06 [8.32-16.68] | 0.72 |
| Lactate (mmol/L) * | | 0.5-2.2 | 1.34 [1.14-1.58] | 1.4 [1.2-1.64] | 0.69 |
| pH | | Male 7.32-7.43, Female 7.35-7.45 | 7.38 [7.36-14.43] | 7.38 [7.36-14.43] | 0.98 |
| SBE (mmol/L) | | -2 -> 3 | 0.6 [-1.05--1.22] | 0 [-1.63--2.36] | 0.61 |
| Platelets (x109/L) | | 140 - 400 | 227.05 [195.62-399.45] | 200 [169-348] | 0.24 |
| PT (secs) | | 9-13 | 14.87 [13.02-26.47] | 15.73 [13.88-28.15] | 0.52 |
| WCC (x109/L) | | 3.5-11 | 14.82 [12.53-25.73] | 12.83 [10.57-21.86] | 0.23 |

Abbreviations: ACTH adrenocorticotropic hormone, ALP alkaline phosphatase, ALT alanine transferase, AST aspartate transferase, BNP brain-natriuretic peptide, FT3 free triiodothyronine, GH growth hormone, HDL high density lipoprotein, PT prothrombin time, SBE standard base excess, T4 thyroxine, TSH thyroid stimulating hormone, WCC white cell count.

Data given as mean [95% CI] except where indicated

*Geometric mean

**Table S6 – Percentage of samples drawn per time point**

|  | **Dexmedetomidine** | **Usual Care** | **p** |
| --- | --- | --- | --- |
| Baseline % (n) | 98.2 (54) | 97.1 (48) | 0.27 |
| Day 1 % (n) | 100.0 (56) | 99.0 (52) | 0.3 |
| Day 3 % (n) | 94.3 (50) | 87.4 (34) | 0.01* |
| Day 5 % (n) | 69.8 (30) | 72.8 (25) | 0.48 |

**Figure S5 - Percentage of samples drawn per time point**

**Table S7 – Medications received in 24hrs prior to randomisation**

|  | **Dexmedetomidine (n=51)** | **Usual Care**  **(n=52)** | **p** |
| --- | --- | --- | --- |
| Beta Blocker % (n) | 21.6 (11) | 19.2 (10) | 0.77 |
| Statin % (n) | 31.4 (16) | 32.7 (17) | 0.89 |
| ACE-I % (n) | 19.6 (10) | 15.4 (8) | 0.57 |
| AII-I % (n) | 9.80 (5) | 26.9 (14) | 0.03* |
| Steroid % (n) | 37.3 (19) | 23.1 (12) | 0.12 |
| TPN % (n) | 9.80 (5) | 9.62 (5) | 0.97 |

Abbreviations: ACE-I angiotensin converting enzyme inhibitor, AII-I angiotensin 2 inhibitor.

**Figure S6 – Medications received in 24hrs prior to randomisation**

**Table S8 – Participants receiving steroids during study period**

|  | **Dexmedetomidine** | **Usual care** | **P** |
| --- | --- | --- | --- |
| Baseline | 37.3%(19/51) | 23.1%(12/52) | 0.14 |
| Day 1 | 31.4%(16/51) | 13.7%(7/51) | 0.033* |
| Day 3 | 27.9%(12/43) | 17.0%(8/47) | 0.31 |
| Day 4 | 27.0%(10/27) | 15.8%(6/32) | 0.23 |

**Figure S7 – Interval from randomisation to sample draw time**
